# Orienting the Interaction Compass: Resource Availability as a Major Driver of Context Dependence

**DOI:** 10.1371/journal.pbio.2000891

**Published:** 2016-10-12

**Authors:** Elizabeth G. Pringle

**Affiliations:** Department of Biology, Program in Ecology, Evolution, and Conservation Biology, University of Nevada, Reno, Nevada, United States of America

## Abstract

Life on earth is enormously diverse, in part because each individual engages in countless interactions with its biotic and abiotic environment during its lifetime. Not only are there many such interactions, but any given interaction of each individual with, say, its neighbor or a nutrient could lead to a different effect on its fitness and on the dynamics of the population of which it is a member. Predicting those effects is an enduring challenge to the field of ecology. Using a simple laboratory system, Hoek and colleagues present evidence that resource availability can be a primary driver of variation between interactions. Their results suggest that a complex continuum of interaction outcomes can result from the simple combined effects of nutrient availability and density-dependent population dynamics. The future is rich with potential to integrate tractable experimental systems like theirs with hypotheses derived from studies of interactions in natural communities.

The science of ecology is plagued or elevated (depending on your perspective) by the tendency for interactions between organisms and their environment to vary in space and time, with differing consequences for behavior, physiology, and/or fitness. This variation, known as context dependence, affects biotic and abiotic interactions alike and frustrates predictive efforts. For example, to determine how herbivory affects a plant population, you have to predict not only (a) how tissue loss will affect plant fitness but also (b) how much tissue will be lost (which depends on the density and identity of herbivores) and (c) how difficult that tissue will be to replace (which depends on resource availability). Herbivore communities and resource availability thus provide the “context” for plant tissue loss, such that herbivory can matter "hardly at all" or "a whole lot" depending on where you are and when you look.

Mutualisms—i.e., interactions with reciprocal fitness benefits (Box 1)—were early poster children for context dependence [1], in part because it seemed difficult to reconcile cooperative behavior with the selective pressure to minimize interaction costs [2]. Such selection can destabilize mutualism by favoring the evolution of exploiters (or "cheaters"), whose effects on their interacting partners are dampened or even reversed (i.e., resulting in parasitism; Box 1). Compounding this paradox still further, some mutualisms occur within a trophic level, where substantial niche overlap between partners also renders them potential competitors. Recent work on microbes nevertheless suggests that mutualism readily evolves between partners at the same trophic level under certain environmental conditions [3]. Work on positive interactions between plants had in fact previously suggested a general "stress gradient hypothesis" for predicting these context-dependent outcomes: interactions should transition from negative to positive along gradients of increasing environmental stress [4]. Although derived from plant community ecology, the stress gradient hypothesis has recently gained traction in diverse mutualisms [5–8]. So far, this concordance is more about pattern than process; elucidating process is, however, ultimately essential to understanding context dependence.

**Box 1. The Interaction Compass**

Interactions are usually defined by the direction in which they affect the interactors, be they species, strains, or individuals. Even as variation in interspecific interactions first came into focus [1,9], it was clear that both the strength and the sign of interactions shifted back and forth along a continuum (Fig 1). The center of the interaction compass (see [10,11]) is sometimes called neutralism, but this box classifies any interaction where a fitness effect does not occur. Although the interaction compass is typically shown with only two species for the purposes of illustration, all species are involved in networks of interactions, and indirect interactions—defined where one species affects another by way of a third species or pathway—are ubiquitous in ecological communities and can rival direct interactions in their strength [e.g., 12]. The variety of terms and their distinct historical origins can lead to some ambiguity, as is the case with mutualism and facilitation [13]. Facilitation does not appear in the interaction compass (Fig 1), but it is associated with some of the earliest research on positive interactions across environmental gradients and with the stress gradient hypothesis in particular [4]. The term arises from 20th-century plant community ecology and refers either to any interaction where one species modifies the environment in a way that is positive for a neighboring species or specifically to positive interactions within a trophic level. Relevant here, until the recent surge of interest in microbe-microbe interactions, the term mutualism typically referred to interactions between trophic levels, where the competition outcome (––) is unlikely because the interactors do not overlap substantially in their niche requirements. It is common to speak instead of the mutualism-parasitism continuum. Although microbes fit perhaps only uncomfortably into the trophic boxes defined on the basis of macroorganism interactions, most cross-feeding mutualisms occur within a trophic level and thus could be thought of as examples of both mutualism and facilitation, with outcomes ranging around the full compass, from mutualism to competition and back to mutualism again.

**Fig 1:**
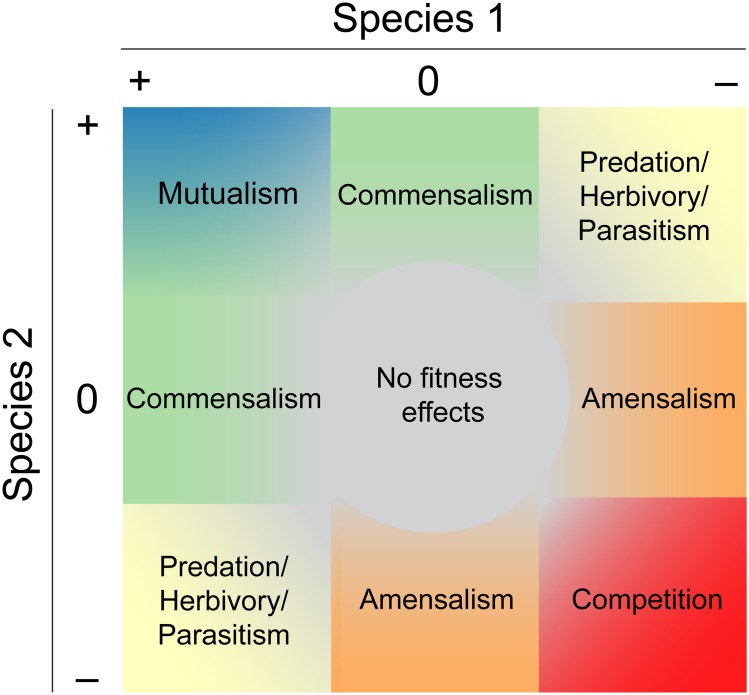
The interaction compass. A two-species interaction is illustrated with the terms defining each of the differently signed outcomes; the signs indicate individual fitness or population growth rate. A positive (+) sign thus indicates a positive effect of the interaction on the individual or population, a zero (0) sign indicates no effect, and a negative (–) sign indicates a negative effect. Moving away from the center increases the magnitude of the net effect of the interaction.

One relatively straightforward path by which an increase in stress can lead to stronger mutualism is when the interaction involves a direct exchange of the environmentally limiting resource. In North American grasslands, grasses associate with arbuscular mycorrhizal fungi, which exchange soil nutrients for carbon fixed by the grass. The fungi can deliver both phosphorus and nitrogen, increasing grass uptake of whichever nutrient is least available in a given soil [5]. Although this seems like a good trick, we cannot characterize the outcome of the grass's interaction with the fungi on the basis of nutrient uptake (the benefit) alone. The delivery of carbon by the grass to the fungi (the cost), and the net balance of trade (benefit−cost), is key. In this example, the grass receives a net benefit (increased biomass) from interacting with the fungi in phosphorus-poor soil but not in nitrogen-poor soil [5]. The fungi thus seem to be parasites in nitrogen-poor soil, but, interestingly, even that interaction is less negative for grasses in soils with less nitrogen [5]. This example suggests potentially broad relevance for the stress gradient hypothesis across the continuum of interaction types (Box 1) (Fig 1) but also highlights how the balance of trade determines ecological outcomes. To predict the outcome of any given interaction, therefore, we need to understand how both the benefits and the costs of interactions depend on an organism's environment. This is a challenging task, requiring integrative understanding of organismal physiology, axes of environmental variation, and the nature of biotic interactions, as well as of the feedbacks between organismal ecology and evolution.

The diversity and experimental tractability of microorganisms, as well as their fundamental role in life on earth, make them appealing systems for studying context dependence in its multiple dimensions. The potential for mutualism among microbes and between microbes and their multicellular hosts is receiving unprecedented attention as the diverse and important roles of the human gut microbiome come into sharp relief. As field-based studies of macroorganism interactions move past the recognition of context dependence to a deliberate focus on its drivers and mechanisms [14], laboratory-based studies of microbes are, in parallel, moving past debates about the "typical" nature of microbial interactions [15,16] to focus on how and why interaction outcomes vary across environmental gradients [3,17].

In this issue of *PLOS Biology*, Hoek and colleagues show that interactions between two cross-feeding yeast strains can transition across nearly a full continuum of outcomes with simple variation in environmental nutrient concentration [18]. Cross-feeding microbes are those with similar metabolic requirements whose metabolic pathways are complementary, either because of a "leaky" byproduct system whereby some metabolites end up in the environment [15] or because of costly, cooperative exchange [19]. In the Hoek et al. study, the investigators used strains of cross-feeding yeast engineered to differ in amino acid production: one strain lacks leucine production but overproduces tryptophan (Leu^−^), and the other lacks tryptophan production but overproduces leucine (Trp^−^). By varying the quantity of leucine and tryptophan in the environment in a constant ratio, the investigators produced a continuum of interaction outcomes, from low-amino-acid environments that exhibit obligate mutualism to high-amino-acid environments that exhibit strong competition. They go on to show that many of these dynamics can be recovered with a remarkably simple model of each strain's population growth, primarily depending only on the quantity of environmental amino acids and the population densities of the two strains. The complete range of empirically determined qualitative change in the interaction is mirrored by this simple model, which suggests that the outcomes of interactions that depend on resource exchange (including most mutualisms! [20]) can be predicted to an impressive degree by measuring the availability of that resource, the population densities of the interacting species, and their intrinsic growth rates.

Returning to our grass-fungi interaction from above, however, we recall that the benefits of interacting (receiving the missing amino acid in the case of these yeasts) are only one side of the coin. The costs of interacting are what underlie the conflicts of interest that threaten mutualism stability and can lead to increasingly negative interactions over evolutionary time. In the Hoek et al. study, the costs of overproducing the amino acid that is consumed by the other strain are modeled only implicitly in the intrinsic growth rate (*r*), which is determined by growing each strain in monoculture with unlimited amino acids. The major discrepancy between their model and the empirical data, however, is that the model incorrectly predicts a much larger range of amino-acid concentrations at which the Trp^−^ strain is expected to outperform the Leu^−^ strain in both monoculture and co-culture. Interestingly, the Trp^−^ strain performs particularly poorly when it is co-cultured with the Leu^−^ strain, except at very high levels of environmental amino-acid availability. It is tempting to speculate that this discrepancy is caused by the model's lack of an explicit density-dependent cost of leucine production, which would be exacerbated when the Leu^−^ strain is performing well. Going forward, it should be possible to merge population dynamic models that include such a cost [21] with an explicit term for resource availability to see how well these models predict dynamics in a variety of empirical systems.

Rapid, ongoing global change presents one compelling reason to determine how resource availability underpins interactions, and the Hoek et al. study also sheds some needed light here. Using their model, they determine distinct early-warning "signatures" of imminent population collapse for co-cultures at very low levels of amino acids (think, e.g., drought), in which both strains go extinct upon the collapse of the obligate mutualism, and at very high levels of amino acids (think, e.g., nutrient pollution), in which competitive exclusion leads to the extinction of the slower growing strain. The collapse of populations engaging in obligate mutualism is predicted when the ratio of the population densities of the two strains becomes stable much more quickly than the total population size (particularly at small population sizes) and vice versa for competitive exclusion. In contrast, in healthy populations, the ratio of the strains and the total population size become stable at approximately equal speeds. As the authors note, this result suggests that we can predict how close one or both interacting species are to extinction by monitoring their comparative population dynamics.

This is an exciting prospect, but how easy is it to monitor the population dynamics of interacting species outside the laboratory? Monitoring populations of long-lived species in the field is inherently difficult, and, historically, less attention has been paid to determining how the environment affects populations of interacting species than to how it affects individual traits and fitness [14]. But wait, you say, surely individual fitness is the driving force behind population dynamics. Well, yes and no. To assess individual fitness, investigators almost always use one or more proxies, including growth, survival, and reproductive biomass. Population-level studies have shown that a given interacting species can have multiple, frequently opposing effects on these different components of their partner's fitness, such that an exclusive focus on any one component can be very misleading [22,23]. In addition, population dynamics depend on the probability of successful offspring recruitment; this probability is critical because it itself is also likely to vary along environmental gradients [14]. Population-level approaches thus deserve explicit focus despite their challenges, and this is one area where field studies can be greatly enhanced by both theory and model systems.

A more serious problem, perhaps, and one that confronts all manner of approaches and systems, is how to scale up from simplified studies of two interacting species to entire ecological communities [24,25]. To use distinct dynamical signatures to predict population collapse, we need to know what other threats a species faces as well as what other opportunities are available. For example, corals exchange nutrients and protection for fixed carbon from their photosynthetic algal symbionts in the genus *Symbiodinium*. There are at least four major clades within *Symbiodinium* that associate with corals, and any given coral can associate with more than one type of algae, either simultaneously or throughout its lifetime. Temperature is an important environmental stress for corals, leading to the well known and increasingly problematic coral bleaching phenomenon, in which corals lose the carbon source provided by their algal symbionts (through loss of the symbionts themselves or of the symbionts' photosynthetic capacity) as the oceans warm. However, some algal symbionts are more thermally tolerant than others, such that corals under temperature stress may lose their association with one symbiont (i.e., collapse of that obligate mutualism) only to gain assocation with a second, more thermally tolerant symbiont (i.e., formation of a different obligate mutualism) [26]. Various alternative responses to environmental stress can be imagined by considering interactions in a community context (Fig 2), and our understanding of these scenarios in well studied field systems should be mined to generate hypotheses about lesser known microbial interaction networks.

**Fig 2:**
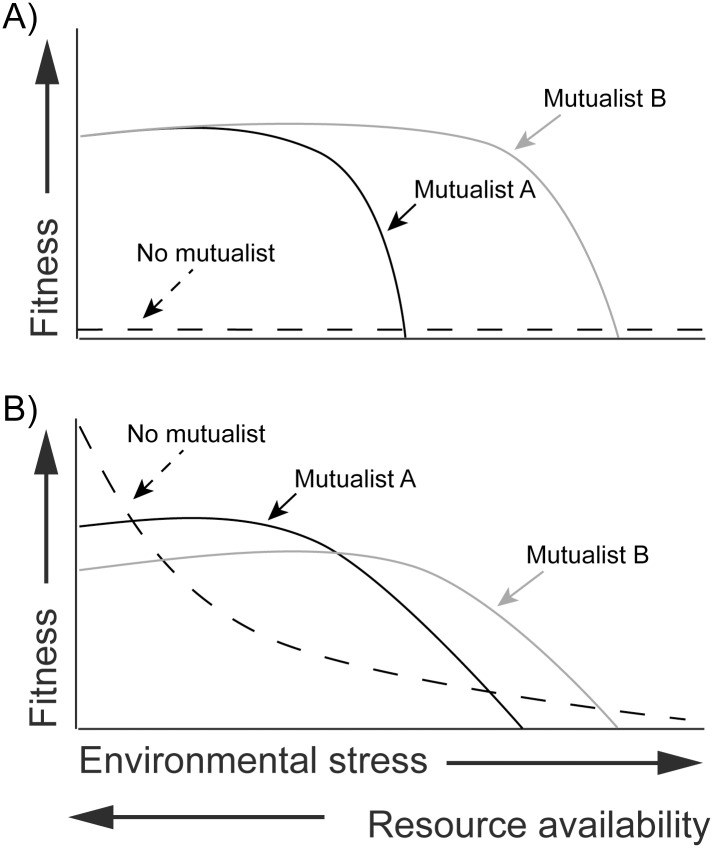
Mutualism in a community context. Multispecies interactions can exhibit a greater variety of outcomes than two-way interactions. (A) An interaction in which the mutualism is always obligate (individuals with no mutualist have zero fitness), but some mutualists are better than others at high environmental stress (e.g., as in the coral-algae example). (B) An interaction in which there are multiple mutualists that vary in their cost to the partner. The costly mutualist is more effective, so Mutualist A increases fitness more than Mutualist B when stress is low to intermediate, but the cost of Mutualist A exceeds its benefit when stress is intermediate to high. In this mutualism, unlike in (A), the partner can exist independently of its mutualists and actually does so with higher fitness when environmental stress is very low (resource availability is very high) or stress is very high (resource availability is very low).

The challenges to elucidating the drivers and mechanisms of context dependence are real, but the work of Hoek and colleagues reminds us that complex outcomes do not necessarily require complicated explanations. The emerging parallels between the burgeoning study of interactions among microbes and the research on species interactions in the macro-world should not be ignored and should, in fact, be leveraged for additional insight. For example, a functional approach is being pursued in both subfields and may increase our ability to generalize across highly diverse systems [24,27], but the context dependence of such functional types themselves [28] must be recognized and investigated in tandem. Simple systems like these cross-feeding yeasts suggest numerous possible future experiments to study what happens when we add dimensions in the environment or the species pool under high levels of experimental control. This study emphasizes the importance of resource availability for orienting the interaction compass.
